# Clinical risk factors for mortality in an analysis of 1375 patients admitted for COVID treatment

**DOI:** 10.1038/s41598-021-02920-w

**Published:** 2021-12-03

**Authors:** Sean A. P. Clouston, Benjamin J. Luft, Edward Sun

**Affiliations:** 1 Stony Brook Medicine, Stony Brook, NY USA; 2 Stony Brook University Hospital, Stony Brook, NY USA; 3 Program in Public Health, Stony Brook Health Sciences Center, Stony Brook University, 101 Nichols Rd., Stony Brook, NY 11794-8338 USA

**Keywords:** Cardiovascular diseases, Infectious diseases, Psychiatric disorders

## Abstract

The goal of the present work was to examine clinical risk factors for mortality in 1375 COVID + patients admitted to a hospital in Suffolk County, NY. Data were collated by the hospital epidemiological service for patients admitted from 3/7/2020 to 9/1/2020. Time until final discharge or death was the outcome. Cox proportional hazards models were used to estimate time until death among admitted patients. In total, all cases had resolved leading to 207 deaths. Length of stay was significantly longer in those who died as compared to those who did not (p = 0.007). Of patients who had been discharged, 54 were readmitted and nine subsequently died. Multivariable-adjusted Cox proportional hazards regression revealed that in addition to older age, male sex, and a history of chronic heart failure, chronic obstructive pulmonary disease, and diabetes, that a history of premorbid depression was a risk factors for COVID-19 mortality (aHR = 2.42 [1.38–4.23] P = 0.002), and that this association remained after adjusting for age and for neuropsychiatric conditions as well as medical comorbidities including cardiovascular disease and pulmonary conditions. Sex-stratified analyses revealed that associations between mortality and depression was strongest in males (aHR = 4.45 [2.04–9.72], P < 0.001), and that the association between heart failure and mortality was strongest in participants aged < 65 years old (aHR = 30.50 [9.17–101.48], P < 0.001). While an increasing number of studies have identified several comorbid medical conditions including chronic heart failure and age of patient as risk factors for mortality in COVID + patients, this study confirmed several prior reports and also noted that a history of depression is an independent risk factor for COVID-19 mortality.

The SARS-CoV-2 pandemic has caused a global emergency causing millions of infections and cases of SARS-CoV-2-related disease (COVID-19) with New York City (NYC) being an early epicenter in the U.S.^1^. SARS-CoV-2 is a highly infectious disease with reports of the mean reproductive rate to range from 1.83 to 2.06 and mean time until death of 17–24 days since symptom onset^2^. Suffolk County, a large suburban commuter community on Long Island where essential workers for NYC often reside, was hit early with large numbers of cases and deaths attributed to COVID-19^3^.

There is a rapidly growing body of evidence examining details of COVID-19’s structure and epidemic dynamics^4^. To determine severity of disease, clinicians rely on an increasing array of indicators of disease severity among infected patients (e.g., cardiac injury^5^ and hypoxia^6^, biomarkers including D-dimer, low systolic blood pressure, and oxygen saturation^7^, or large vessel strokes in younger patients^8^) to determine severity of COVID-19. Recently, there has been increasing attention to the impact of COVID-19 and functional changes to the nervous system^9^ as indicated by changes to neuropsychiatric symptomatology^10^. Notably, infection with COVID-19 has now been linked with increased depressive symptoms^11^, anosmia^12^, cerebrovascular neuropathology^13^, as well as peripheral immunologic changes and increased risk of stroke^14^.

While clinical markers of COVID-19 severity and course are critical to case management, premorbid risk factors for severe disease can be helpful when trying to determine the risk due to exposure and when trying to manage resources for treatment. Known risk factors include aging^15^ as well as a range of aging-related conditions^16,17^. However, many pre-existing conditions have not survived age adjustment^18^. As noted in one meta-analysis, risk factors that have been consistently identified in addition to age are cardiovascular disease, hypertension, diabetes, and respiratory disease^19^. Yet, there remains a lack of clarity from these studies with the largest meta-analysis to date (N = 17,860,001) identifying older age, male sex, and severe obesity as risk factors for mortality^20^.

Noting that research prior to the COVID-19 epidemic suggests that patients with elevated depressive symptoms have significant neuro- and systemic immune dysfunction and activation^21^, we hypothesized that COVID-19 might be more severe in patients with a history of depression. The objective of the current study was to examine clinical risk factors for mortality in an inpatient sample admitted for COVID-19 treatment. We hypothesized that chronic conditions related to more rapid aging would be associated with increased risk of COVID-related mortality after adjusting for age, sex, and other confounders.

## Methods

Stony Brook University Hospital (SBUH) is the largest medical center on Long Island, NY and manages the only Level I Adult Trauma Center. SBUH began monitoring its first confirmed COVID-19 in early March. The current study examined outcomes among COVID + patients admitted from 3/7 to 5/15/2020. Outcomes were censored at death or on 9/1/2020 to allow time for cases to resolve. Patients who died in the emergency room prior to admission or within one day of admission were not included in multivariable analyses.

Patients who died prior to admission ranged in age from 46 to 101 years, none were depressed, six were female, and had on average 1.75 comorbidities including COPD and atrial fibrillation but not depression or chronic heart failure. In cases of readmissions for patients who had been discharged, the date of first admission was retained and, if applicable, the new date of discharge or death was recorded. Age-stratified subgroup analyses were completed, and sensitivity analyses were completed examining risk factors only for patients whose treatment was completed.

### Measures

The outcome was days until death or final discharge was the outcome among inpatients admitted to the hospital during the first wave for care for COVID-19 infection. COVID-19 status was confirmed by a polymerase chain reaction (PCR) positive test.

Pre-existing conditions were recorded at intake by emergency medicine clinicians. While intake forms recorded all previously diagnosed conditions, only comorbidities that occurred frequently enough to identify statistically significant relationships were examined. For example, pregnancy status, mental disabilities, and cause-specific cancers were excluded because of low prevalence rates. Specifically, we recorded pre-existing diagnoses of (1) neurological or psychiatric disorders including generalized anxiety, major depression, and dementia; (2) cardiovascular conditions including atrial fibrillation, cardio-arterial disease, chronic heart failure, hyperlipidemia, hypertension, and stroke; (3) pulmonary conditions including asthma, and chronic obstructive pulmonary disease; and (4) other diseases including obesity, diabetes mellitus, and gastroesophageal reflux disease, chronic kidney disease, and all-cause cancer. Demographic variables included age in years, and sex. Age was entered in years, rather than by 5-year age groups, because age categorization usually results in loss of information and attribution of age variance to aging-related conditions. We also identified patients with no premorbid conditions. Sensitivity analyses additionally considered accounting for the number of comorbid conditions, which in this population ranged from 0 to 19 (mean = 3.13 [2.82]) but since these analyses did not identify significant correlations, this variable was dropped from models presented.

While the goal of the present study was not to study the effects of in-hospital treatments, some have noted that intubated patients were at increased risk of mortality^22^. Mechanical ventilation was most likely to be used among individuals with the most severe disease in the early weeks of disease management prior to April 15, 2020. Since then, intubation rates have fallen drastically. Since mortality rates, and vulnerability to COVID-related mortality may differ in intubated patients, we initially examined risk factors in intubated and non-intubated patients. We compared risk factors in patients who had been admitted prior to April 15th when ventilation was still being used regularly *versus* thereafter when ventilation was much less common. To examine the importance of adjusting for intubation, we also conducted multivariable analyses adjusting for whether an individual had been intubated during their inpatient stay. In the analyses presented, intubation was included as a covariate because it independently influenced the risk of mortality but was unlikely to be the mechanism through which premorbid risk factors might influence mortality risk. Sensitivity analyses examined to determine whether excluding intubation status changed results and results were briefly reported.

*Ethics:* The Internal Review Board at Stony Brook University approved this study (IRB#1,586,703). All study activities were performed under exemption 4iii of the revised common rule; a waiver for the need for informed consent was granted.

### Statistical analysis

Patient characteristics were reported using means and standard deviations or percentages (%). Welch’s t-tests and χ^2^ tests were used to complete unadjusted analyses. Multivariable-adjusted Cox proportional hazards regression was used to estimate time until death among all admitted patients when adjusting for age^23^. The exact method was used to account for ties^24^. Proportional hazards assumptions were tested. Model concordance (Harrel’s C) was reported to determine predictive accuracy; C ranges from 0.50 (poor predictor) through 0.70–0.80 (very good accuracy) to 1.00 (perfect prediction). To provide a predictive model we reduced the number of variables included in the final model to those that were consistent when adjusting for age/sex and when adjusting for all covariates and reported those with p-value < 0.10.

All analyses were completed using Stata 17.1/SE [StataCorp].

### Power analysis

Power simulations determined that at least 1,300 patients were required to achieve multivariable-adjusted power estimates (α = 0.05, power = 0.80); a priori projections completed in April indicated May 15th as study closing date.

### Ethics approval and consent to participate

The study was reviewed and approved by the Stony Brook Ethics Review Board; informed consent was not required because this was a secondary analysis of clinical data to help inform healthcare practices.

## Results

The current study examined outcomes among COVID + patients (N = 1,375; 207 deaths) who were admitted alive from 3/7 to 5/15/2020 (mean length of stay = 13.12 [SD = 18.07] days). Outcomes were censored at death or on 9/1/2020 to allow time for patients to resolve. At the time of analysis, all cases had been discharged (n = 1168) or had died (n = 207). Length of stay was significantly longer in those who died (16.54 days) as compared to those who did not (12.51 days; difference = 4.02 days, p = 0.003). Of patients who had been discharged, 54 patients were later readmitted (4.58% [3.46–5.93]) and nine (16.67% [7.92–29.29]) subsequently died.

COVID + patients had a range of premorbid physical conditions including atrial fibrillation, cardio-arterial disease, chronic obstructive pulmonary disease, heart failure, hyperlipidemia, hypertension, and stroke (Table 1). The mortality rate was 1.15% [1.00–1.31] per day. Age was a strong predictor of mortality in all models with each additional year of age associated with 4.54% [3.57–5.53] increase in the risk of mortality in these models. Age alone was a very good predictor of the risk of mortality in this population (C = 0.74). The predictive power was very good in females (C = 0.77) and males (C = 0.72), and in both individuals with any comorbidities (C = 0.72) and among those with without any comorbidities (C = 0.81).

**Table 1:** Patient characteristics for 1375 patients admitted to Stony Brook University Hospital between March–May 15th, 2020.

Patient characteristic	Admitted patients (N = 1375)	Patients who survived (N = 1,168)	Patients who expired (N = 207)	Survived *versus* expired
Age, mean (SD)	60.13 (19.55)	58.02 (19.55)	72.03 (14.69)	< 0.001
Female	589 (42.84)	518 (44.35)	71 (34.3)	0.004
No comorbidities	215 (15.64)	197 (16.87)	18 (8.7)	0.002
Intubated during stay	215 (15.64)	108 (9.25)	107 (51.69)	< 0.001
**Cause-specific comorbidities**				
Anxiety	35 (2.55)	28 (2.4)	7 (3.38)	0.424
Asthma	85 (6.18)	71 (6.08)	14 (6.76)	0.814
Atrial fibrillation	100 (7.27)	72 (6.16)	28 (13.53)	< 0.001
Cancer	68 (4.95)	55 (4.71)	13 (6.28)	0.252
Cardio-arterial disease	170 (12.36)	125 (10.7)	45 (21.74)	< 0.001
Chronic kidney disease	29 (2.11)	23 (1.97)	6 (2.9)	0.248
Chronic obstructive pulmonary disease	87 (6.33)	58 (4.97)	29 (14.01)	< 0.001
Dementia	67 (4.87)	49 (4.2)	18 (8.7)	0.006
Depression	59 (4.29)	44 (3.77)	15 (7.25)	0.031
Diabetes mellitus	343 (24.95)	271 (23.2)	72 (34.78)	< 0.001
Gastroesophageal reflux disease	122 (8.87)	99 (8.48)	23 (11.11)	0.178
Heart failure	65 (4.73)	40 (3.42)	25 (12.08)	< 0.001
Hyperlipidemia	279 (20.29)	219 (18.75)	60 (28.99)	0.001
Hypertension	571 (41.53)	456 (39.04)	115 (55.56)	< 0.001
Obesity	361 (26.25)	311 (26.63)	50 (24.15)	0.413
Stroke	67 (4.87)	46 (3.94)	21 (10.14)	< 0.001

Characteristics report N (%) unless otherwise noted. SD: Standard deviation; N: number of patients; % percentage. P-values report results from unadjusted t-tests in the case of age in years, and chi-squared tests for all other characteristics.

Early efforts relied on ventilator usage to treat patients, resulting in large differences in treatment course so that 23.36% (n = 103) of all admitted patients placed on ventilation March–April 15th, 2020, *versus* only 11.99% (n = 112) after April 15th (a difference of 11.36%, p < 0.001). Mortality rates were not higher in patients admitted in March, when ventilation use was common, when compared to the rest of the period upon adjusting for age (aHR = 1.15, 95% CI [0.85–1.56], p = 0.355). Yet, mortality rates in April/May did increase when accounting for ventilator usage (aHR = 1.41 [1.03–1.92], p = 0.031), potentially indicating that the shift away from ventilation was not always optimal for individual patients.

Age/Sex-adjusted models identified intubation, having any comorbidities, atrial fibrillation, cardio-arterial disease, chronic obstructive pulmonary disease, dementia, depression, diabetes, gastroesophageal reflux disease, heart failure, hyperlipidemia, hypertension, and stroke were possible risk factors for COVID-19 mortality (Table 2). Multivariable-adjusted Cox proportional hazards regression (Model 2) revealed that only intubation status, and a history of chronic obstructive pulmonary disease, depression, diabetes, and heart failure remained statistically significant risk factors for COVID + mortality. These results were supported in a reduced model excluding variables that were not statistically significant in both models (Table 3).

**Table 2:** Age/sex-adjusted and multivariable-adjusted hazards ratios and 95% Confidence intervals derived from Cox proportional hazards regression models examining predictors of mortality in COVID + patients.

	Model 1: age/sex-adjusted			Model 2: multivariable adjusted		
Patient characteristic	HR	95% CI	P	aHR	95% CI	P
No comorbidities	0.80	(0.49–1.32)	0.385	0.87	(0.49–1.54)	0.638
Intubated	2.33	(1.72–3.17)	< 0.001	2.55	(1.87–3.50)	< 0.001
**Pre-existing medical comorbidities**						
Anxiety	1.59	(0.74–3.43)	0.237	1.16	(0.50–2.67)	0.730
Asthma	0.90	(0.52–1.57)	0.720	0.80	(0.45–1.43)	0.456
Atrial fibrillation	1.23	(0.81–1.86)	0.339	1.03	(0.65–1.62)	0.908
Cancer	0.96	(0.54–1.70)	0.875	1.10	(0.60–2.01)	0.756
Cardio-arterial disease	1.01	(0.71–1.44)	0.945	0.90	(0.62–1.30)	0.582
Chronic kidney disease	1.18	(0.51–2.70)	0.703	1.06	(0.42–2.69)	0.899
Chronic obstructive pulmonary disease	1.77	(1.18–2.65)	0.006	1.61	(1.04–2.48)	0.031
Dementia	1.03	(0.62–1.71)	0.918	1.05	(0.50–2.20)	0.899
Depression	2.04	(1.18–3.52)	0.011	2.27	(1.26–4.07)	0.006
Diabetes mellitus	1.38	(1.03–1.84)	0.032	1.35	(0.99–1.86)	0.061
Gastroesophageal reflux disease	1.28	(0.82–2.00)	0.277	1.15	(0.71–1.86)	0.582
Heart Failure	2.36	(1.51–1.56)	< 0.001	2.67	(1.64–1.63)	< 0.001
Hyperlipidemia	1.04	(0.76–1.42)	0.811	0.98	(0.70–1.38)	0.929
Hypertension	1.06	(0.79–1.42)	0.705	1.03	(0.74–1.43)	0.871
Obesity	1.12	(0.81–1.55)	0.509	0.91	(0.64–1.29)	0.596
Stroke	1.35	(0.84–2.17)	0.217	1.26	(0.62–2.57)	0.519

HR: Unadjusted hazards ratios; aHR: Multivariable adjusted hazards ratio; 95% CI: 95% Confidence Interval. The proportional hazards assumptions were met in all models.

**Table 3:** Multivariable-adjusted hazards ratios and 95% Confidence intervals from a reduced Cox proportional hazards regression models in the whole sample.

Patient characteristics	aHR	95% CI	P
Age, years	1.05	(1.04–1.06)	< 0.001
Male	1.31	(0.96–1.78)	0.083
Intubated	2.49	(1.83–3.39)	< 0.001
Chronic obstructive pulmonary disease	1.54	(1.02–2.34)	0.042
Depression	2.42	(1.38–4.23)	0.002
Diabetes mellitus	1.39	(1.04–1.86)	0.028
Heart failure	2.68	(1.69–4.26)	< 0.001

aHR: Multivariable-adjusted hazards ratio; 95% CI: 95% Confidence Interval.

Harrel’s Concordance = 0.78.

### Additional analyses

Stratified subgroup analyses (Table 4) in older (≥ 65 years) *versus* younger patients (< 65 years) were substantively similar showing for example that depression was a risk factors for mortality in older patients. Subgroup analyses suggested that the association between depression and mortality was stronger in males than females.

**Table 4:** Multivariable-adjusted hazards ratios and 95% Confidence intervals derived from Cox proportional hazards regression models stratified by sex (Panel A) and by age (Panel B) examining predictors of mortality in COVID + patients.

Panel A: sex-stratified	Male			Female		
Patient characteristics	aHR	95% CI	P	aHR	95% CI	P
Age, years	1.04	(1.03–1.06)	< 0.001	1.06	(1.03–1.08)	< 0.001
Chronic obstructive pulmonary disease	1.80	(1.07–3.02)	0.026	1.41	(0.70–2.83)	0.340
Depression	4.45	(2.04–9.72)	< 0.001	1.55	(0.69–3.47)	0.290
Diabetes mellitus	1.17	(0.80–1.70)	0.413	1.92	(1.16–3.16)	0.011
Heart failure	4.73	(2.53–8.85)	< 0.001	1.66	(0.82–3.35)	0.160
Intubated	3.09	(2.07–4.60)	< 0.001	2.20	(1.24–3.90)	0.007

Panel B: age-stratified	Under 65 years			65 and older		
Patient characteristics	aHR	95% CI	P	aHR	95% CI	P
Male	1.33	(0.79–2.56)	0.386	1.43	(1.00–2.00)	0.051
Chronic obstructive pulmonary disease	1.26	(0.30–5.36)	0.755	1.45	(0.94–2.24)	0.097
Depression	3.79	(0.83–17.37)	0.086	2.63	(1.43–4.84)	0.002
Diabetes mellitus	0.97	(0.53–1.75)	0.915	1.47	(1.04–2.07)	0.029
Heart failure	30.50	(9.17–101.48)	< 0.001	2.31	(1.41–3.78)	0.001
Intubated	11.34	(5.03–25.57)	< 0.001	1.39	(0.96–2.02)	0.078

aHR: multivariable-adjusted hazards ratio; 95% CI: 95% Confidence Interval.

Sensitivity analyses did not identify factors that differed between intubated and non-intubated patients, and risk factors did not differ in the era of ventilation as compared to post-ventilation (after April 15th). Additionally, models considering the number of comorbid conditions did not change interpretation as compared to the models presented. Analyses used to examine the benefit of including variables such as anxiety that were consistent and showed near-significant results across models did not provide results that were statistically significant in the reduced model, so they were not incorporated into the final predictive model. Sensitivity analyses examining differences when excluding intubation status revealed similar results showing older age and a history of diabetes, depression, and heart failure were all risk factors for mortality in COVID patients.

## Discussion

The causes of mortality in COVID + patients is a topic of intense interest for researchers worldwide. This study sought to determine whether a history of depression would be a risk factors for mortality in COVID + patients. In the present study, we relied on a large cohort of patients admitted for COVID-19 in New York to determine that in addition to older age and male sex, a history of depression and heart failure were risk factors for COVID-19 mortality. Subgroup analyses clarified that depression was a risk factor for COVID mortality in older patients, but that depression was most predictive in men as compared to women.

COVID-19 causes older infected individuals to experience more severe disease and large increases in risk of mortality^15,25^. Vulnerability to COVID-19 is a topic of intense interest that is important to individuals who may have an array of premorbid conditions and who may be making efforts to avoid infection. Prior work has highlighted a relatively large array of aging-related conditions such as diabetes and hypertension as potential risk factors for COVID-19. The current study supports earlier work suggesting that age is a substantively important risk factor for COVID-related mortality.

Individuals are increasingly being asked to return to work or else to justify staying home with many workers relying on comorbid conditions as a reason to justify working from home. The current study suggested that not all comorbidities confer increased vulnerability to COVID-19 mortality. However, we also found that lacking any comorbidities was not associated with reduced hazards of mortality among COVID + patients. Our study suggests that while several comorbid conditions were associated with increased risk of COVID-related mortality in a medium-sized hospital in a large suburban community, more research is warranted to better understand the mechanisms linking these risk factors to increased risk of mortality in these patients.

Our finding that depression is a risk factor for mortality in COVID + patients supports prior analyses in a large population database^26^. Mechanisms for such a relationship may include the potential for neuro- and systemic immune dysfunction that is common among individuals with depression^21^. Indeed, patients with depressive symptoms have heightened levels of circulating proinflammatory cytokines including interleukin-6^27^, and heightened production of pro-inflammatory cytokines in response to stressors^28^. This may be particularly important in the pathogenesis of COVID-19 given the purported crucial role that interleukin-6 plays in some initial reports of adverse outcomes related to COVID-19.

Prior reports have suggested that hypertension and diabetes may both be risk factors for COVID-19 mortality^29^. The nature of such relationships is still subject to some debate, but appears to be relatively consistent^30,31^. In one early study (n = 140), hypertension was described as being the most common comorbidity with a prevalence of 30%^32^. One problem is that hypertension and diabetes are high-prevalence age-related conditions. The Centers for Disease Control and Prevention^33^ estimate the prevalence of diabetes and hypertension in New York State to be 11.0% and 29.4% respectively, with estimates increasing to 24.1% and 55.6% respectively in respondents aged 65 and older. In the current study (mean age 59.78), COVID + patients had diabetes and hypertension at prevalence rates in this cohort that closely matched prevalence in the population highlighting the critical need for age-adjustment in research on studies examining predictors of COVID-19 mortality. Results from a meta-analysis suggest that the high level of association originally reported was likely an over-estimate, due to a lack of adjustment for other factors^34^. In this study, we found that hypertension was a risk factor for COVID mortality in bivariate analyses, but multivariable adjustment reduced this association. One interpretation of this is that the method of adjustment for age, sex, and other confounders may explain differences between studies. An alternative interpretation may be that the types of treatments being used for hypertension in this population were more frequently angiotensin-converting enzyme inhibitors, which may be more effective in reducing the excess risk carried by hypertension^35^. Additional research is needed to clarify the nature of this association.

Heart failure is a complex aging-related condition characterized by reduced functional capacity that carries reduced quality of life, increased healthcare usage, and high risk of mortality^36^. Individuals with heart failure are at increased risk of dying from a range of conditions including from stroke and anemia, as well as from lung, liver, and kidney diseases^37^. Heart failure is a common comorbidity in atrial fibrillation, a condition that often causes heart failure and also carries high risk of stroke^38^. Together, these analyses support the increasingly common view that COVID-19 may cause a clotting disorder that accelerates mortality due to cardiovascular and cerebrovascular causes.

Analyses adjusted for intubation status. Intubation was a critical factor that, early in the pandemic, may have increased mortality rates in those who were intubated. In our analyses, we adjusted for intubation status because of the iatrogenic effects that it played in higher mortality rates. While being associated with the severity of disease, we felt it was not the likely mechanism through which depression or other comorbidities might impact the risk of mortality but also felt that it might independently hasten mortality.

### Limitations

Though being among the earliest studies to identify pre-existing conditions that increase risk of mortality in COVID + patients, this study is also limited in focusing on patients admitted to a single hospital on Long Island, NY. Information about race/ethnicity and socioeconomic status were not recorded. A number of studies have noted neuropsychiatric changes among COVID + patients^10^. NY. The current study did not seek to determine indicators of cardiovascular, pulmonary, or cerebrovascular indicators of COVID-19 severity but instead sought to identify patients for whom exposure to SARS-CoV-2 may be more deadly. Analyses did not identify dementia as a risk factor for mortality even though Alzheimer’s disease, a main cause of dementia, is a neuroinflammatory condition that causes elevated levels of c-reactive protein and cytokines commonly related to COVID-19. However, since individuals with Alzheimer’s disease and vascular dementia are often cared for in nursing homes, where many outpatient deaths are known to have occurred, this effect may be underestimated in studies such as this one that rely on inpatient samples. In the case that encephalopathy is present, but may not always become severe, future research is warranted to examine the potential for a lasting post-COVID encephalopathy among survivors. Major depression is a heterogeneous condition with a range of potential etiologies; we were unable here to examine whether subsyndromal depression or specific depressive symptoms were attributable for the increased risk identified herein. Due to limitations in the available data, we could not adjust for socioeconomic or racial/ethnic disadvantage here. Prior work suggests that these may play a significant role both in the risk of infection and in the risk of mortality. Further efforts are needed to characterize these risk factors for disease.

### Implications

There is increasing agreement that COVID-19 causes changes in the central nervous system^9^ and that neuropsychiatric effects may be important when characterizing COVID-19 severity^39^. There is also increasing recognition that these changes may carry long-term behavioral and functional consequences^40^. The current study extends prior studies to suggest that those with pre-existing depression may also be at higher risk of experiencing the most severe forms of the disease.

## Data Availability

All data include sensitive private health information. As such, data are being maintained by the PI but are also available to other researchers who are interested via the TriNetX data housing service. Methods: All methods were performed in accordance with the relevant guidelines and regulations.
